# The single recombinant *M. tuberculosis* protein DPPD provides enhanced performance of skin testing among HIV-infected tuberculosis patients

**DOI:** 10.1186/s13568-020-01068-6

**Published:** 2020-07-31

**Authors:** Roberto Badaro, Bruna. A. S. Machado, Malcolm S. Duthie, C. A. Araujo-Neto, D. Pedral-Sampaio, Maria Nakatani, Steven G. Reed

**Affiliations:** 1 SENAI Institute of Innovation (ISI) in Advanced Health Systems (CIMATEC ISI SAS), University Center SENAI/CIMATEC, National Service of Industrial Learning–SENAI, Salvador, Brazil; 2 Department of Medicine, Federal University of Bahia, Salvador, Brazil; 3 HDT Bio Corp, 1616 Eastlake Ave E, Seattle, WA 98102 USA

**Keywords:** Tuberculosis, Mycobacteria, Skin, T cell, Diagnosis

## Abstract

Diagnostic testing for *M. tuberculosis* infection has advanced with QuantiFERON and GeneXpert, but simple cost-effective alternatives for widespread TB screening has remained elusive and purified protein derivative (PPD)-based tuberculin skin testing (TST) remains the most widely used method. PPD-based tests have reduced performance, however, in BCG vaccinees and in individuals with immune deficiencies. We compared the performance of skin testing with the recombinant DPPD protein against that of a standard PPD-based skin test. Our data indicates similar performance of DPPD and PPD (r^2^ = 0.7689) among HIV-negative, active TB patients, all of whom presented greater than 10 mm induration following administration. In contrast to results demonstrating that PPD induced indurations greater than 5 mm (i.e., the recommended threshold for positive results in this population) in only half (19 of 38) of the HIV positive TB patients, 89.5% (34 of 38) of these participants developed indurations greater than 5 mm when challenged with DPPD. Importantly, none of the patients that were positive following PPD administration were negative following DPPD administration, indicating markedly improved sensitivity of DPPD among HIV-infected individuals. Our data indicate that DPPD has superior performance in skin testing than the current TST standard.

## Key points

- Recombinant DPPD provides a simple skin test for detecting TB patients.
- Recombinant DPPD performs similarly to crude PPD among HIV negative TB patients.
- Diagnostic capacity among HIV-positive individuals is improved with DPPD.

## Introduction

Tuberculosis (TB) was the primary cause of 1.3 million deaths in 2017, making it the second most common cause of death from infectious diseases (WHO 2018a). Estimates are that nearly 2 billion people latently infected with *Mycobacterium tuberculosis* and 5–10% of infected individuals will progress to TB disease during their lifetime (Getahun et al. 2015; Houben and Dodd 2016; WHO 2018a). *M. tuberculosis* infection is especially problematic in immune compromised individuals that have an increased likelihood of progression to disease such that TB is among the leading cause of death in HIV/AIDS patients. Accordingly, the World Health Organization (WHO) considers TB a global health emergency.

A wide variety of strategies are utilized to diagnose TB. Indirect methods such as chest X-rays are commonly used to confirm symptoms. Direct detection systems have included culture of *M. tuberculosis* from sputum samples, and modern technologies such as nucleic acid amplification tests (i.e., GeneXpert) have revolutionized the diagnosis of active TB by allowing faster generation of more sensitive and specific results that improve patient care (Boehme et al. 2011; Nicol et al. 2011). Given difficulties in sputum collection etc., these tests still have limited overall sensitivity, however, and even with generous subsidies and concessional pricing they remain too expensive for routine use in the majority of resource-limited settings.

Indirect detection tests that use anti-*M. tuberculosis* immune responses as a readout can be conducted in decentralized research settings and permit widespread surveillance programs. Assays that measure interferon (IFN)-γ secretion upon incubation of unfractionated blood with Mtb proteins (IFN-γ release assays; IGRA) have been developed (Arenas Miras Mdel et al. 2013; Arias Guillen 2011; Rangaka et al. 2012). The cost and modest availability in most countries with limited resources have constrained the use of IGRA and prohibit their widespread use (Morrison et al. 2008; Owusu-Edusei et al. 2017; Pai et al. 2014; Rangaka et al. 2012). Even in the United States, where 2013 medical expenditures for TB-specific tests among the employer-based privately insured population were estimated to be $53.0 million, private insurance claims data relating to TB testing indicated that IGRA were used far less often than tuberculin skin tests (TST) (13.7% versus 86.3%, respectively). TST were not only the most commonly used test, they were by far the least expensive ($9) diagnostic strategy used (Owusu-Edusei et al. 2017). Thus, both cost and practicality mean that skin tests are the more likely diagnostic strategy to be used, and many TB experts have favored the development of a skin test that could be more accurate for the diagnosis of active TB and LTBI (Pai et al. 2016; Rangaka et al. 2012; Rose et al. 1995).

Clinicians have great familiarity with skin testing and TST has been the standard tuberculosis screening/surveillance tool for decades (Bass 2003; Snider 1982), generating results by inducing a measurable delayed type hypersensitivity (DTH) reaction at the site of intradermal injection of purified protein derivative (PPD; tuberculin). PPD is a precipitate of species-nonspecific molecules from filtrates of sterilized tuberculoid organism cultures and many varieties of PPD are actually in use. PPD-S2, the current US standard that was developed to address the eventual depletion of PPD-S (Villarino et al. 2000), is used in the commercially available Aplisol^®^ and Tubersol^®^ (Jensen et al. 2005). On June 6, 2019, Centers for Disease Control (CDC) announced an anticipated 3–10 month shortage of Aplisol, one of two purified-protein derivative (PPD) tuberculin antigens licensed in the US (2019). Tubersol, the other PPD licensed test, has also experienced regular shortages (CDC 2013b). Officials in 56% US jurisdictions have reported shortages of PPD TST antigen solutions in health departments (10 Tubersol only, four Aplisol only, and 15 both) to the extent that routine activities had been threatened or already curtailed (CDC 2013a).

We previously published preliminary results of a skin test for *M. tuberculosis* infection that was based on a novel recombinant protein produced from a gene unique to MTB. This protein, named DPPD on the basis of the first 4 amino acids of its N-terminus sequence, elicited delayed-type hypersensitivity (DTH) in 100% of *M. tuberculosis*-infected guinea pigs but not in animals sensitized with other *Mycobacteria* representative of the genus (Coler et al. 2000). This small proof-of-concept study indicated that intradermal DPPD skin testing detected individuals exposed to MTB with higher sensitivity and specificity than had previously been reported with the TST using PPD (Campos-Neto et al. 2001).

Here we report the findings from an open label evaluation of the sensitivity and specificity of DPPD among groups for whom testing results are traditionally poor, including those with prior BCG vaccination, active tuberculosis, and concurrent HIV infection.

## Materials and methods

### Study design

This was a prospective open label clinical trial conducted in Salvador, Brazil. The study population was recruited at two sites: The University Hospital Professor Edgard Santos (HUPES) and the AIDS Reference Center (CREAIDS) of the state of Bahia, Brazil.

### Study population

A total of 375 individuals were screened, with 173 enrolled for skin test evaluations. Exclusions were predominantly made on the basis of lack of BCG scar and lack of microbial confirmation of TB. Participants were enrolled into one of 3 groups following their provision of informed consent (Table 1). Group 1 (Healthy Controls with Prior BCG Vaccination) consisted of 95 persons > 18 years of age who reported no respiratory symptoms, had a documented BCG vaccination scar on the right arm and were confirmed to be HIV negative. Group 2 (Active TB, without HIV infection; n = 40) and Group 3 (Active TB, with HIV infection; n = 38) included adult patients either without or with HIV infection who were treated for presumptive active TB. Chest radiographs were performed on all study participants, and patients presenting with palpable lymph nodes at study entry underwent needle aspiration or lymph node dissection to allow histopathologic evaluation of tuberculous involvement. Active TB was defined as fulfilling the following criteria: clinical signs and symptoms of pulmonary disease consistent with active tuberculosis; a chest radiograph prior to treatment consistent with active pulmonary TB; and a sputum sample that was positive for *M. tuberculosis* by either Ziehl–Neelsen staining or culture from Lowenstein-Jensen medium (Additional file 1: Figure S1). Active TB was further stratified into 3 clinical categories: (A) Pulmonary TB (PTB), defined by a chest radiograph consistent with TB; (B) Lymph node disease (LD), defined as a lymph node with histologic findings of acid-fast bacilli or growth of MTB from culture; and C) Disseminated TB (DTB), defined by presence of miliary pulmonary nodules and hepatosplenomegaly. Blood samples were collected from HIV-positive participants in Group 3 to quantify T cell subsets.

**Table 1:** Demographics of study groups

	Healthy	TB disease	
	BCG immunized	HIV negative	HIV positive
N	95	40	38
Gender			
Male	42	18	32
Female	53	22	6
Age (years)			
Mean	29.8	31.2	39.9
Min–max	18–47	18–54	19–64
Clinical presentation (n)			
Cavitary	–	20	4
Disseminated	–	9	33
Lymphadenopathy	–	31	37

### Antigen preparation and skin testing

Skin testing was conducted with either recombinant DPPD or Tubersol PPD Tuberculin (Connaught Laboratories Ltd, Willowdale, Ontario, Canada). Testing with DPPD was conducted following preparation as previously described (Campos-Neto et al. 2001). Briefly, the recombinant DPPD protein was diluted with sterile PBS containing Tween 80 (0.0005%) as a stabilizer and 0.28% phenol as a preservative (i.e., the same contents as Tubersol PPD Tuberculin). The final solution was sterile filtered (0.2 m) and dispensed in 5 ml aliquots in sterile vials. Sterility and general safety testing were performed as specified by the regulations of the United States Food and Drug Administration. To perform the skin tests, 0.1 ml of each antigenic solution (with 1 µg of DPPD bioequivalent to 5 TU of Tubersol PPD Tuberculin) was injected intradermal (Mantoux technique) on each forearm. PPD was injected in the right arm and DPPD was injected in the left arm. Reactions were read by trained healthcare workers 48–72 h after administration, with induration measured in millimeters (mm) using standard methods (Dacso 1990). Results are expressed in mm of transverse diameter of induration. Consistent with the instructions of the Tubersol product insert, induration ≥ 10 mm was considered positive in individuals from high-risk areas (i.e. the recruitment site used here) and induration ≥ 5 mm was considered positive in HIV-positive individuals.

### Statistical analyses

Statistical analyses were conducted with GraphPad Prism (GraphPad Software, San Diego, CA). Based on the standard interpretation of r^2^ (coefficient of determination) values, correlation was considered weak when 0.3 < r^2^ < 0.5, moderate when 0.5 < r^2^ < 0.7, and strong when r^2^ > 0.7 were found.

## Results

### Performance of DPPD in skin testing among target populations

**Fig. 1:**
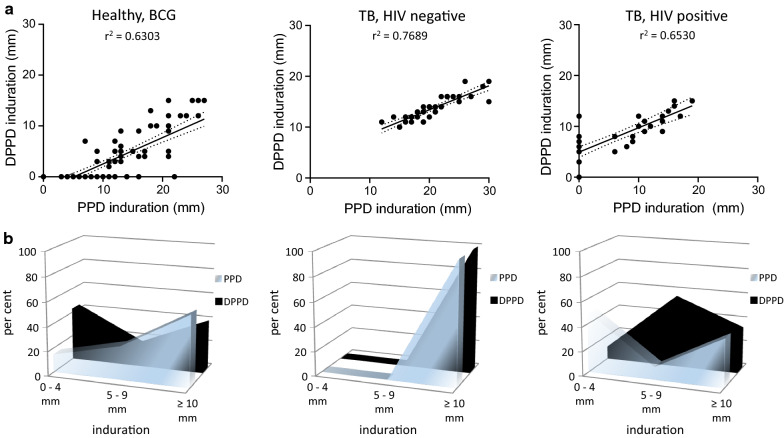
Skin testing with recombinant proteins. Healthy, BCG immunized individuals (n = 95) and TB patients, either negative (n = 40) or positive (n = 38) for HIV infection, were intradermally inoculated with 5 TU of Tubersol PPD Tuberculin or the bioequivalent 1 µg of DPPD. Induration was measured 24 h later. Results were determined to be positive when, consistent with CDC guidelines, induration > 10 mm in HIV negative individuals and > 5 mm in HIV positive individuals. In A, each dot represents an individual data point, while the solid line represents the correlation of results and the black dotted lines represent the 95% confidence intervals. The r^2^ value is indicated in the text insert. In B, per cent of each group falling within the indicated induration range is plotted

We then evaluated the diagnostic capacity of DPPD as a skin test antigen by comparing against that of the gold standard, PPD. Indurations following PPD administration were generally greater than those observed following DPPD administration (Fig. 1). Although there was a moderate correlation between PPD and DPPD results (r^2^ = 0.6303), an induration of greater than 10 mm was observed in a lower proportion of healthy participants (i.e., those without active TB but with previous BCG vaccination) following administration of DPPD than following injection with PPD (13.6% versus 57.9%, respectively) (Fig. 1a). Thus, DPPD appears to provide more specific results than PPD.

Among active TB patients, irrespective of if TB disease was of a cavitary, disseminated or lymphadenopathic presentation, all 40 HIV-negative individuals were positive at > 10 mm induration following administration of either PPD or DPPD (Fig. 1b) and there was strong correlation between test results (r^2^ = 0.7689). These data indicate that DPPD performance is similar to that of PPD for the identification of HIV negative, TB patients.

Among patients with active TB and HIV co-infection, there was again a moderate correlation between the magnitude of response elicited by either PPD or DPPD (r^2^ = 0.6530). In contrast to results generated with PPD demonstrating that only half (19 of 38) of the HIV positive TB patients developed indurations greater than 5 mm (i.e., the recommended threshold for positive results in this population), in this group 89.5% (34 of 38) of participants developed indurations greater than 5 mm when challenged with DPPD. Importantly, none of the patients that were positive following PPD administration were negative following DPPD administration, indicating the improved sensitivity of DPPD among HIV-infected individuals.

### Performance of skin testing relative to CD4 T cell status in HIV-infected individuals

**Fig. 2:**
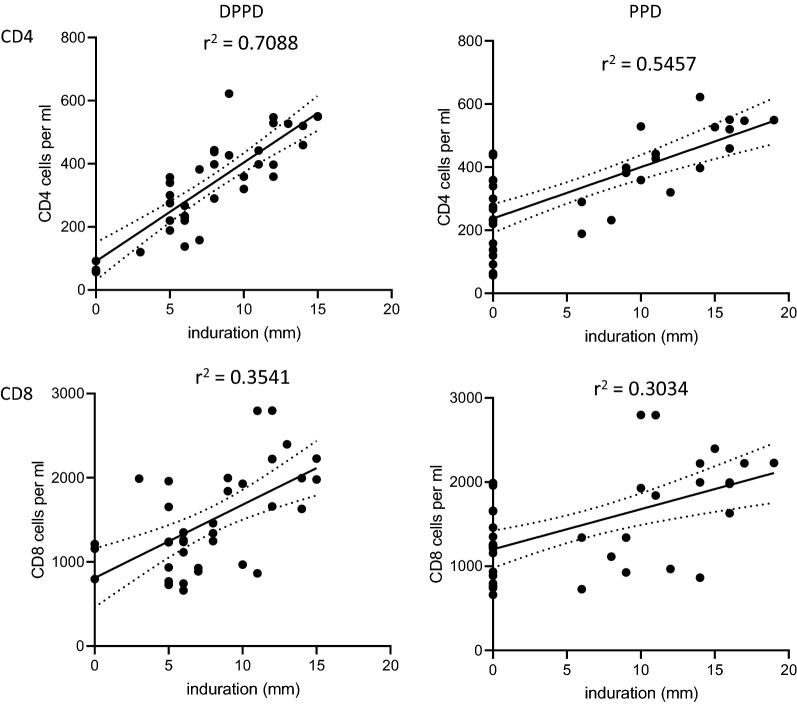
Correlation of T cell numbers with antigen-induced induration. CD4 and CD8 T cell counts were measured for TB patients that were HIV positive (n = 38) and are plotted against the skin induration measured 24 h after they were intradermally inoculated with either 5 TU of Tubersol PPD Tuberculin or the bioequivalent 1 µg of DPPD. Each dot represents an individual data point, while the solid line represents the correlation of results and the black dotted lines represent the 95% confidence intervals. The r^2^ value is indicated in the text insert. In B, per cent of each group falling within the indicated induration range is plotted

Given that a delayed type hypersensitivity response is elicited following intradermal administration of antigen, we hypothesized that CD4 T cell counts would be a major determinant of the induration size following skin testing in HIV positive TB patients. Accordingly, when we compared induration size against CD4 cell counts, we observed a strong correlation following testing with DPPD and a moderate correlation following testing with PPD (r^2^ = 0.7088 and 0.5457, respectively) (Fig. 2a). Induration size demonstrated only a weak correlation with CD8 cell counts (r^2^ = 0.3541 and 0.3034 for DPPD and PPD, respectively) (Fig. 2b). These data substantiate the performance of DPPD and indicate retained performance in HIV-infected individuals even with declining CD4 T cell counts.

## Discussion

Although TST remains the most widely used method for diagnosis of *M. tuberculosis* infection, PPD-based tests are limited by the reduced performance in populations that have been vaccinated with BCG and in individuals with immune deficiencies. Here we compared the performance of skin testing with recombinant DPPD against that of a standard PPD-based skin test in persons previously vaccinated with BCG and in HIV-infected patients presenting with clinical signs and symptoms consistent with active TB. Our data indicate that, relative to PPD, a skin test using DPPD can not only better distinguish TB among BCG vaccinees but has improved performance among HIV-infected TB patients. It is our belief that this advantage, along with the practical consideration that decentralized use of skin tests is attainable because they not require a laboratory component, provide a competitive advantage for the extended use of a DPPD-based skin test.

We developed a skin test based on the recombinant DPPD protein not only to address concerns surrounding sustainable, consistent production of PPD but also to provide a cost-effective alternative. PPD is a precipitate of species-nonspecific molecules from filtrates of sterilized tuberculoid organism cultures and many varieties of PPD are actually in use. Outside of North America, PPD RT23 is the most widely used PPD product, but PPD RT23 Mexico is used in Latin America, PPD-s is used in Japan and PPD-L is used in Russia (Comstock et al. 1964; Kimura et al. 2005; Rangel-Frausto et al. 2001; Starshinova et al. 2018; Yang et al. 2012). PPD-S2, the current US standard that was developed to address the eventual depletion of PPD-S, is used in the commercially available Aplisol^®^ and Tubersol^®^ (Jensen et al. 2005; Villarino et al. 2000). Although results of testing with Aplisol^®^ and Tubersol^®^ were comparable with that of the original PPD-S, shifting use between Tubersol^®^ and Aplisol^®^ resulted in aberrations for reasons that are unclear (Gillenwater et al. 2006; Mehta et al. 2009; Villarino et al. 1999). Mass spectrometry and molecular analyses identified over one hundred proteins from four different PPD, revealing that the heat shock proteins GroEl, GroEs, DnaK, and HspX that are conserved amongst most mycobacterial species contribute to roughly 60% of the total PPD protein content (Borsuk et al. 2009; Cho et al. 2012). It is believed that this wide mixture of mycobacterial proteins contributes to the inability of PPD-based TST to distinguish *Mtb* infection from either exposure to non-tuberculous mycobacteria or vaccination with BCG (Farhat et al. 2006; Huebner et al. 1993). The DPPD protein, encoded by *Rv0061*, is unique to the *Mtb* complex and is recognized by a strong DTH response in *Mtb*-infected guinea pigs and tuberculosis patients (Campos-Neto et al. 2001; Coler et al. 2000; Liu et al. 2004). Data indicate that guinea pigs sensitized with crude mycobacterial antigen mixtures from each of *M. bovis*–BCG, *M. avium*, *M. kansasii*, *M. fortuitum*, *M. gordonae*, *M. chelonae*, *M. scrofulaceum*, *M. smegmatis*, *M. terrae*, and *M. vaccae* do not exhibit DTH responses against DPPD (Coler et al. 2000). The data in BCG-immunized individuals reported here supports improved specificity of DPPD relative to PPD. Previous evaluation of 270 healthy individuals similarly found that DPPD was less likely to elicit induration than PPD-S (Tubersol^®^) (Campos-Neto et al. 2001). It should be noted, however, that neither of these studies could discern if a positive DPPD result in healthy individuals was due to latent tuberculosis or a stronger BCG-induced response. This could potentially be delineated by incorporating a demographically similar BCG “naïve” population (such as individuals in the United States), but this introduces social and geographical (environmental mycobacteria) variables that makes them less than ideal comparators.

The use of TST for the diagnosis of active disease in HIV-infected individuals has been controversial with previous studies reporting reduced sensitivity in this population (Goldstein et al. 1994; Graham et al. 1992; Johnson et al. 1992; Lifson et al. 1993; Mamani et al. 2013; Whittle et al. 1993). It is therefore noteworthy that we also found that the DPPD appeared to be more sensitive for the detection of active TB in HIV-infected individuals than PPD. Magnitude of response to DPPD correlated strongly with CD4 T cell counts of HIV-infected patients. Even in the patients with CD4 T cell counts below 200 cells per ml (therefore fulfilling the definition of AIDS), 3 of 6 had positive induration of ≥ 5 mm in response to DPPD. This contrasted with the 1 of 6 that were positive upon evaluation with PPD. Expanded testing among individuals with defined CD4 T cell counts is required to validate this observation.

Modelling for elimination programs of diseases in which many people harbor infection without evidence of clinical symptoms emphasize the importance of detecting and treating these individuals (Cameron et al. 2016; Dye et al. 2013; Keeler et al. 2006). Unless the chain of *M. tuberculosis* transmission is disrupted the global TB elimination targets are unlikely to be reached and, accordingly, the identification and treatment of latent TB infection (LTBI) is a critical component within the WHO End TB Strategy (Lonnroth and Raviglione 2016; Uplekar et al. 2015; WHO 2018b). In addition to a clear economic advantage, it is our belief that skin testing with DPPD presents a practical advantage over the IGRA and nucleic acid amplification tests (NAAT) have been developed (Arenas Miras Mdel et al. 2013; Arias Guillen 2011; Rangaka et al. 2012). For example, a skin test is well suited for use in decentralized field studies or surveillance programs where even rudimentary laboratory support is lacking.

Our study does have some important limitations, perhaps foremost the evaluation of a relatively small number of patients in one geographic location. Therefore, building upon the data we present here, future evaluations of the DPPD-based skin test should include larger numbers of individuals at differing risk for LTBI. Testing in settings with differing exposure to environmental mycobacteria is also necessary, as well as direct comparison against other assays such as QuantiFERON and GeneXpert. In summary, the clinical data presented here demonstrates that fewer BCG immunized individuals respond to recombinant DPPD than crude PPD, patients with active TB respond strongly to DPPD skin test, and unlike PPD, the response to DPPD is preserved in HIV-infected TB patients. These results not only indicate that recombinant DPPD elicits a DTH response but also, most importantly, that it could replace the antigenic mixture represented by PPD while simultaneously providing improved performance in the HIV-affected communities that are highly at-risk for TB disease. This represents a major advance over the current strategy of skin testing with PPD that is used for TB detection in most countries.

## Supplementary information

**Additional file 1: Figure 1.** Active TB was defined by clinical signs and symptoms of pulmonary disease consistent with active tuberculosis and a chestradiograph prior to treatment consistent with active pulmonary TB. Among such patients, a sputum sample was evaluated for the presence of M. tuberculosisby either Ziehl–Neelsenstaining (bacilloscopy) or outgrowth from culture in Lowenstein-Jensenmedium (culture). Per cent positive among HIV-negative and HIC-positive TB patients is shown.

## Data Availability

Supporting data can be provided from the authors upon reasonable request

## Abbreviations

AIDS: Acquired immune deficiency syndrome
DTH: Delayed type hypersensitivity
HIV: Human immunodeficiency virus
IFN: Interferon
IGRA: IFNγ release assay
LTBI: Latent *Mycobacterium tuberculosis* infection
NAAT: Nucleic acid amplification tests
PPD: Purified protein derivative
TB: Tuberculosis
TST: Tuberculin skin test
WBA: Whole blood assay
WHO: World Health Organization

## References

[CR1] Arenas Miras Mdel M, Hidalgo Tenorio C, Jimenez Alonso J (2013). Tuberculosis in patients with systemic lupus erythematosus: Spain’s situation. Reumatol Clin.

[CR2] Arias Guillen M (2011). Advances in the diagnosis of tuberculosis infection. Arch Bronconeumol.

[CR3] Bass JB (2003). How good is the tuberculin skin test?. Infect Control Hosp Epidemiol.

[CR4] Boehme CC, Nicol MP, Nabeta P, Michael JS, Gotuzzo E, Tahirli R, Gler MT, Blakemore R, Worodria W, Gray C, Huang L, Caceres T, Mehdiyev R, Raymond L, Whitelaw A, Sagadevan K, Alexander H, Albert H, Cobelens F, Cox H, Alland D, Perkins MD (2011). Feasibility, diagnostic accuracy, and effectiveness of decentralised use of the Xpert MTB/RIF test for diagnosis of tuberculosis and multidrug resistance: a multicentre implementation study. Lancet.

[CR5] Borsuk S, Newcombe J, Mendum TA, Dellagostin OA, McFadden J (2009). Identification of proteins from tuberculin purified protein derivative (PPD) by LC-MS/MS. Tuberculosis (Edinb).

[CR6] Cameron MM, Acosta-Serrano A, Bern C, Boelaert M, den Boer M, Burza S, Chapman LA, Chaskopoulou A, Coleman M, Courtenay O, Croft S, Das P, Dilger E, Foster G, Garlapati R, Haines L, Harris A, Hemingway J, Hollingsworth TD, Jervis S, Medley G, Miles M, Paine M, Picado A, Poche R, Ready P, Rogers M, Rowland M, Sundar S, de Vlas SJ, Weetman D (2016). Understanding the transmission dynamics of *Leishmania donovani* to provide robust evidence for interventions to eliminate visceral leishmaniasis in Bihar, India. Parasit Vectors.

[CR7] Campos-Neto A, Rodrigues-Junior V, Pedral-Sampaio DB, Netto EM, Ovendale PJ, Coler RN, Skeiky YA, Badaro R, Reed SG (2001). Evaluation of DPPD, a single recombinant *Mycobacterium tuberculosis* protein as an alternative antigen for the Mantoux test. Tuberculosis (Edinb).

[CR8] CDC (2013). Extent and effects of recurrent shortages of purified-protein derivative tuberculin skin test antigen solutions - United States, 2013. MMWR Morb Mortal Wkly Rep.

[CR9] CDC (2013). National shortage of purified-protein derivative tuberculin products. MMWR Morb Mortal Wkly Rep.

[CR10] CDC (2019). Nationwide shortage of tuberculin skin test antigens: CDC recommendations for patient care and public health practice. MMWR Morb Mortal Wkly Rep.

[CR11] Cho YS, Dobos KM, Prenni J, Yang H, Hess A, Rosenkrands I, Andersen P, Ryoo SW, Bai GH, Brennan MJ, Izzo A, Bielefeldt-Ohmann H, Belisle JT (2012). Deciphering the proteome of the in vivo diagnostic reagent “purified protein derivative” from *Mycobacterium tuberculosis*. Proteomics.

[CR12] Coler RN, Skeiky YA, Ovendale PJ, Vedvick TS, Gervassi L, Guderian J, Jen S, Reed SG, Campos-Neto A (2000). Cloning of a *Mycobacterium tuberculosis* gene encoding a purifed protein derivative protein that elicits strong tuberculosis-specific delayed-type hypersensitivity. J Infect Dis.

[CR13] Comstock GW, Edwards LB, Philip RN, Winn WA (1964). A Comparison in the United States of America of two tuberculins, Ppd-S and Rt 23. Bull World Health Organ.

[CR14] Dacso CC (1990) Skin Testing for Tuberculosis, vol 47. Clinical methods: the history, physical, and laboratory examinations, Boston 21250045

[CR15] Dye C, Glaziou P, Floyd K, Raviglione M (2013). Prospects for tuberculosis elimination. Annu Rev Public Health.

[CR16] Farhat M, Greenaway C, Pai M, Menzies D (2006). False-positive tuberculin skin tests: what is the absolute effect of BCG and non-tuberculous mycobacteria?. Int J Tuberc Lung Dis.

[CR17] Getahun H, Matteelli A, Chaisson RE, Raviglione M (2015). Latent *Mycobacterium tuberculosis* infection. N Engl J Med.

[CR18] Gillenwater KA, Sapp SC, Pearce K, Siberry GK (2006). Increase in tuberculin skin test converters among health care workers after a change from Tubersol to Aplisol. Am J Infect Control.

[CR19] Goldstein S, Perlman DC, Salomon N (1994). Two-stage tuberculin skin testing in an HIV-infected population: a preliminary report. Mt Sinai J Med.

[CR20] Graham NM, Nelson KE, Solomon L, Bonds M, Rizzo RT, Scavotto J, Astemborski J, Vlahov D (1992). Prevalence of tuberculin positivity and skin test anergy in HIV-1-seropositive and -seronegative intravenous drug users. JAMA.

[CR21] Houben RM, Dodd PJ (2016). The global burden of latent tuberculosis infection: a re-estimation using mathematical modelling. PLoS Med.

[CR22] Huebner RE, Schein MF, Bass JB (1993). The tuberculin skin test. Clin Infect Dis.

[CR23] Jensen PA, Lambert LA, Iademarco MF, Ridzon R (2005). Guidelines for preventing the transmission of *Mycobacterium tuberculosis* in health-care settings, 2005. MMWR Recomm Rep.

[CR24] Johnson MP, Coberly JS, Clermont HC, Chaisson RE, Davis HL, Losikoff P, Ruff AJ, Boulos R, Halsey NA (1992). Tuberculin skin test reactivity among adults infected with human immunodeficiency virus. J Infect Dis.

[CR25] Keeler E, Perkins MD, Small P, Hanson C, Reed S, Cunningham J, Aledort JE, Hillborne L, Rafael ME, Girosi F, Dye C (2006). Reducing the global burden of tuberculosis: the contribution of improved diagnostics. Nature.

[CR26] Kimura M, Comstock GW, Mori T (2005). Comparison of erythema and induration as results of tuberculin tests. Int J Tuberc Lung Dis.

[CR27] Lifson AR, Watters JK, Thompson S, Crane CM, Wise F (1993). Discrepancies in tuberculin skin test results with two commercial products in a population of intravenous drug users. J Infect Dis.

[CR28] Liu C, Flamoe E, Chen HJ, Carter D, Reed SG, Campos-Neto A (2004). Expression and purification of immunologically reactive DPPD, a recombinant *Mycobacterium tuberculosis* skin test antigen, using *Mycobacterium smegmatis* and *Escherichia coli* host cells. Can J Microbiol.

[CR29] Lonnroth K, Raviglione M (2016). The WHO’s new End TB Strategy in the post-2015 era of the Sustainable Development Goals. Trans R Soc Trop Med Hyg.

[CR30] Mamani M, Majzoobi MM, Torabian S, Mihan R, Alizadeh K (2013). Latent and active tuberculosis: evaluation of injecting drug users. Iran Red Crescent Med J.

[CR31] Mehta SR, MacGruder C, Looney D, Johns S, Smith DM (2009). Differences in tuberculin reactivity as determined in a veterans administration employee health screening program. Clin Vaccine Immunol.

[CR32] Morrison J, Pai M, Hopewell PC (2008). Tuberculosis and latent tuberculosis infection in close contacts of people with pulmonary tuberculosis in low-income and middle-income countries: a systematic review and meta-analysis. Lancet Infect Dis.

[CR33] Nicol MP, Workman L, Isaacs W, Munro J, Black F, Eley B, Boehme CC, Zemanay W, Zar HJ (2011). Accuracy of the Xpert MTB/RIF test for the diagnosis of pulmonary tuberculosis in children admitted to hospital in Cape Town, South Africa: a descriptive study. Lancet Infect Dis.

[CR34] Owusu-Edusei K, Winston CA, Marks SM, Langer AJ, Miramontes R (2017). Tuberculosis test usage and medical expenditures from outpatient insurance claims data, 2013. Tuber Res Treat.

[CR35] Pai M, Denkinger CM, Kik SV, Rangaka MX, Zwerling A, Oxlade O, Metcalfe JZ, Cattamanchi A, Dowdy DW, Dheda K, Banaei N (2014). Gamma interferon release assays for detection of *Mycobacterium tuberculosis* infection. Clin Microbiol Rev.

[CR36] Pai M, Behr MA, Dowdy D, Dheda K, Divangahi M, Boehme CC, Ginsberg A, Swaminathan S, Spigelman M, Getahun H, Menzies D, Raviglione M (2016). Tuberculosis. Nat Rev Dis Primers.

[CR37] Rangaka MX, Wilkinson KA, Glynn JR, Ling D, Menzies D, Mwansa-Kambafwile J, Fielding K, Wilkinson RJ, Pai M (2012). Predictive value of interferon-gamma release assays for incident active tuberculosis: a systematic review and meta-analysis. Lancet Infect Dis.

[CR38] Rangel-Frausto MS, Ponce-De-Leon-Rosales S, Martinez-Abaroa C, Haslov K (2001). Tuberculosis and tuberculin quality: best intentions, misleading results. Infect Control Hosp Epidemiol.

[CR39] Rose DN, Schechter CB, Adler JJ (1995). Interpretation of the tuberculin skin test. J Gen Intern Med.

[CR40] Snider DE (1982). The tuberculin skin test. Am Rev Respir Dis.

[CR41] Starshinova A, Zhuravlev V, Dovgaluk I, Panteleev A, Manina V, Zinchenko U, Istomina E, Pavlova M, Yablonskiy P (2018). A comparison of intradermal test with recombinant tuberculosis allergen (diaskintest) with other immunologic tests in the diagnosis of tuberculosis infection. Int J Mycobacteriol.

[CR42] Uplekar M, Weil D, Lonnroth K, Jaramillo E, Lienhardt C, Dias HM, Falzon D, Floyd K, Gargioni G, Getahun H, Gilpin C, Glaziou P, Grzemska M, Mirzayev F, Nakatani H, Raviglione M, for WsGTBP (2015). WHO’s new end TB strategy. Lancet.

[CR43] Villarino ME, Burman W, Wang YC, Lundergan L, Catanzaro A, Bock N, Jones C, Nolan C (1999). Comparable specificity of 2 commercial tuberculin reagents in persons at low risk for tuberculous infection. JAMA.

[CR44] Villarino ME, Brennan MJ, Nolan CM, Catanzaro A, Lundergan LL, Bock NN, Jones CL, Wang YC, Burman WJ (2000). Comparison testing of current (PPD-S1) and proposed (PPD-S2) reference tuberculin standards. Am J Respir Crit Care Med.

[CR45] Whittle H, Egboga A, Todd J, Morgan G, Rolfe M, Sabally S, Wilkins A, Corrah T (1993). Immunological responses of Gambians in relation to clinical stage of HIV-2 disease. Clin Exp Immunol.

[CR46] WHO (2018). Global Tuberculosis Report 2018.

[CR47] WHO (2018). Latent tuberculosis infection: updated and consolidated guidelines for programmatic management.

[CR48] Yang H, Kruh-Garcia NA, Dobos KM (2012). Purified protein derivatives of tuberculin–past, present, and future. FEMS Immunol Med Microbiol.

