# Supplementary material for: The single recombinant M. tuberculosis protein DPPD provides enhanced performance of skin testing among HIV-infected tuberculosis patients
Source: AMB Express. 2020 Jul 31;10:133. doi: 10.1186/s13568-020-01068-6 (PMC7394993; doi:10.1186/s13568-020-01068-6)
Supplement: Supplementary file 1 — Additional file 1: Figure 1. Active TB was defined by clinical signs and symptoms of pulmonary disease consistent with active tuberculosis and a chestradiograph prior to treatment consistent with active pulmonary TB. Among such patients, a sputum sample was evaluated for the presence of M. tuberculosisby either Ziehl–Neelsenstaining (bacilloscopy) or outgrowth from culture in Lowenstein-Jensenmedium (culture). Per cent positive among HIV-negative and HIC-positive TB patients is shown. [file 13568_2020_1068_MOESM1_ESM.pptx]

## Slide 1
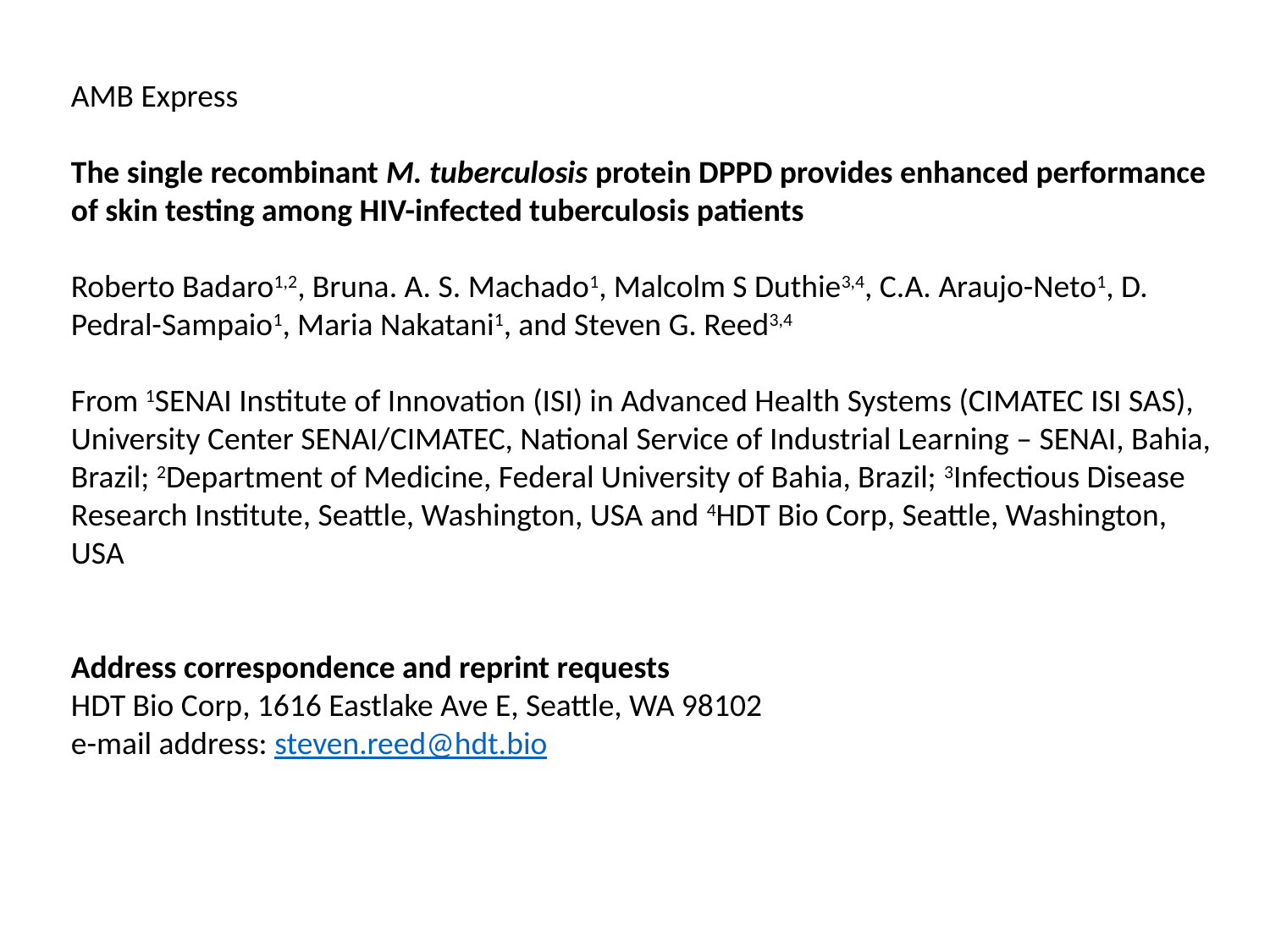

AMB Express
The single recombinant M. tuberculosis protein DPPD provides enhanced performance of skin testing among HIV-infected tuberculosis patients
Roberto Badaro1,2, Bruna. A. S. Machado1, Malcolm S Duthie3,4, C.A. Araujo-Neto1, D. Pedral-Sampaio1, Maria Nakatani1, and Steven G. Reed3,4
From 1SENAI Institute of Innovation (ISI) in Advanced Health Systems (CIMATEC ISI SAS), University Center SENAI/CIMATEC, National Service of Industrial Learning – SENAI, Bahia, Brazil; 2Department of Medicine, Federal University of Bahia, Brazil; 3Infectious Disease Research Institute, Seattle, Washington, USA and 4HDT Bio Corp, Seattle, Washington, USA
Address correspondence and reprint requests
HDT Bio Corp, 1616 Eastlake Ave E, Seattle, WA 98102
e-mail address: steven.reed@hdt.bio

## Slide 2
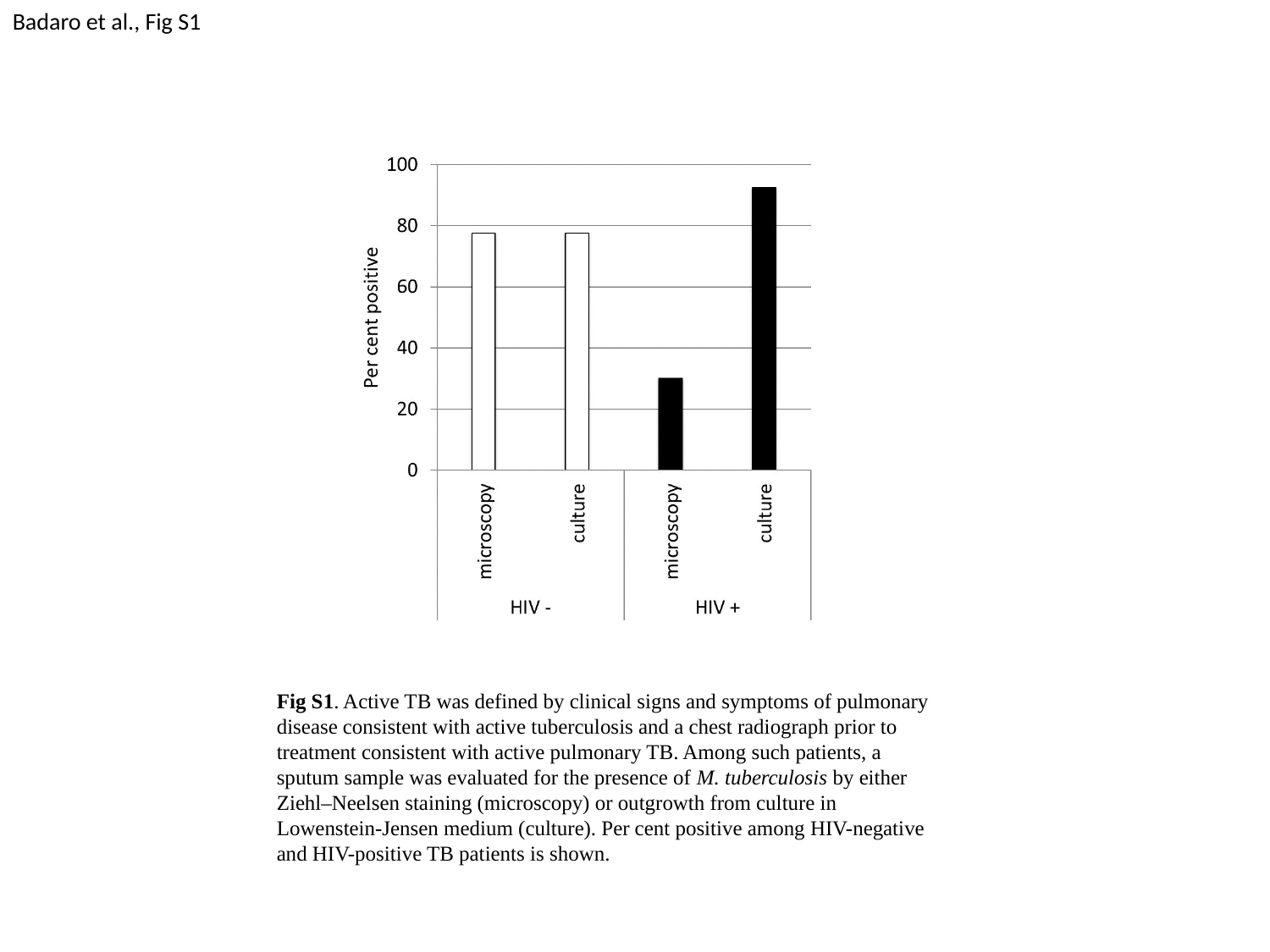

Badaro et al., Fig S1
Fig S1. Active TB was defined by clinical signs and symptoms of pulmonary disease consistent with active tuberculosis and a chest radiograph prior to treatment consistent with active pulmonary TB. Among such patients, a sputum sample was evaluated for the presence of M. tuberculosis by either Ziehl–Neelsen staining (microscopy) or outgrowth from culture in Lowenstein-Jensen medium (culture). Per cent positive among HIV-negative and HIV-positive TB patients is shown.
